# Loneliness in the Era of COVID-19

**DOI:** 10.3389/fpsyg.2020.02219

**Published:** 2020-09-18

**Authors:** Eric D. Miller

**Affiliations:** Department of Psychology, Kent State University, East Liverpool, OH, United States

**Keywords:** COVID-19, social connectedness, loneliness, well-being, mental health

COVID-19 is proving to be a particularly cruel disease not just because of its pathophysiology but also due to its potentially devastating consequences for engendering loneliness. From the outset of the pandemic, we have learned of countless individuals having to die alone or loved ones not being able to grieve by providing burial services (Miller, 2020). This disease holds particularly dire consequences for many populations—most notably the elderly and those with compromised health conditions but also minorities and the homeless; these groups have also faced unique difficulties in contending with loneliness well before this crisis (Rokach, 2019). For as much attention that this disease has rightfully focused on the elderly, individuals can experience different developmental challenges with loneliness throughout the lifespan including adolescence and young adulthood (Luhmann and Hawkley, 2016)—and, indeed, there is already evidence of heightened psychological problems amongst Chinese youth in the wake of this pandemic (Liang et al., 2020). Accordingly, it will be important for psychologists to assess how age-based threats to loneliness evolve in the era of COVID-19.

Social distancing and isolation are critical to preventing the transmission of this highly contagious virus; yet, these are acts that are intrinsically linked with various adverse psychological effects including loneliness and adherence to these sorts of strategies are likely to decrease over time (Armitage and Nellums, 2020; Galea et al., 2020). Given the great reliance on technology during this time, it has been suggested that devices like smartphones can help combat isolation and loneliness during the pandemic particularly among the elderly (Banskota et al., 2020). However, there is a rather conflicting literature as to how Internet-based and social media usage impacts loneliness (Miller, 2018). For instance, Kim (2020) found that social media discussions about COVID-19 were less likely to feature uncivil comments amongst South Korean users with larger social networks. In short, it would be inaccurate to presume that technology provides an absolute clear means of minimizing feelings of loneliness during this crisis.

Even before the pandemic, the World Health Organization declared that social disconnection was a major public health crisis and there is growing concern that the lonely and socially isolated may face heightened morbidity and mortality risks including suicide as a result of this crisis (Courtet et al., 2020). Much more research needs to be done with respect to possible direct or indirect effects from this pandemic that have either created newly found feelings of loneliness or reduced previous such feelings. On one hand, in the wake of this crisis, consistent with basic themes from both terror management theory and attachment theory, a fear of loss of loved ones and love itself should produce a fundamental sense of fear so as to potentially bolster earlier bonds (Steele, 2020). Yet, we are facing what many are terming alarming rises of upwards of 10–20% increased year-to-year rates of domestic violence in select U.S. cities in the immediate aftermath of the crisis (Boserup et al., 2020). More generally, this pandemic may adjust our appraisals of others as they relate to our perceptions of loneliness as it has also served as a reminder of the importance of maintaining health and the fragility of life. Accordingly, it is possible that this crisis may cause individuals to reevaluate aspects of their lives that have contributed to prior perceptions of loneliness. Though, in this era of social distancing and quarantining, quite notably, the sheer act of being alone is not inherently tantamount to producing loneliness (Russell et al., 2012) nor does the physical company of others (such as spouses) inherently prevent loneliness either (Moorman, 2016).

This pandemic will also likely cause us to reflect about our physical environments in a more thoughtful way and this too will have significant relevance to the study of loneliness. A classic paper from Milgram (1970) examined the irony of how urban life can actually allow individuals to feel disconnected from others. Indeed, even though much of the industrialized world is largely urban, such environments can pose challenges to loneliness due to their design and the psychological effects of dense living (Imrie, 2018). The need to address these challenges has likely grown given the newfound perils of physical proximity to others that is largely synonymous with urban life. And, though it may not necessarily be tied to loneliness experienced vis-à-vis close others, feeling connected to nature appears to be associated with a larger connection for humanity and others (Moreton et al., 2019). Since the original COVID-19 source almost certainly was a bat and the point of transmission from animal to human likely occurred at the Wuhan Seafood Market, this experience should force us to realize that in our interdependent world, our actions have clear global consequences and it is critical to have health policies with environmental regulations. In doing so, while it is fair to critique governmental policies or responses that may have contributed to this crisis, it is also important to not do so in a way that further foments racism or stigmatization (Ang, 2020) as these conditions are also associated with the promotion of loneliness.

Over the course of human history, individuals have borne witness to various atrocities and disasters such as war, genocide, and pandemics—and individuals and society generally adapt to these conditions. However, though there remains some debate whether loneliness should be viewed as a modern epidemic, Alberti (2019) contends that it should be looked at it this way when considered through a historical lens and particularly since in the twenty-first century, it is often intertwined by broader social, economic, and political crises. The COVID-19 pandemic features all of these aforesaid crises (Miller, 2020). Though COVID-19 may hold some unique consequences for perceptions of loneliness, so does the potential for technology to help foster self-care during this crisis (Saltzman et al., 2020). Some of the common reasons why loneliness may occur (such as distance from close others or feeling alienated) or ways we cope with loneliness (such as increased activity) may be hindered due to the nature of the pandemic; likewise, pre-existing mental, and physical health risks of loneliness may be exacerbated too during this pandemic. But, indeed, there are effective techniques (that do not necessarily require an online component) such as increased self-reflection and acceptance that can potentially be utilized during this crisis (Sønderby and Wagoner, 2013). Psychological theory, practice, and research must accordingly work to address what will likely be an ever-burgeoning loneliness crisis in the coming years as a result of this pandemic.

## Author Contributions

The author confirms being the sole contributor of this work and has approved it for publication.

## Conflict of Interest

The author declares that the research was conducted in the absence of any commercial or financial relationships that could be construed as a potential conflict of interest.
